# Dermatomyositis Associated with Myelofibrosis following Polycythemia Vera

**DOI:** 10.1155/2017/9091612

**Published:** 2017-06-04

**Authors:** Naomi Fei, Sarah Sofka

**Affiliations:** Department of Internal Medicine, West Virginia University Hospital, 1 Medical Center Dr., Morgantown, WV 26505, USA

## Abstract

Dermatomyositis (DM) is a unique inflammatory myopathy with clinical findings of proximal muscle weakness, characteristic rash, and elevated muscle enzymes. The association of DM and malignancy, most commonly adenocarcinoma, is well known. There have been few case reports of primary myelofibrosis associated with DM. We present the case of a 69-year-old male with a history of polycythemia vera (PV) who developed proximal muscle weakness, dysphagia, and rash. He was found to have elevated creatinine kinase and skin biopsy was consistent with DM. Due to persistent pancytopenia a bone marrow biopsy was performed and showed postpolycythemic myelofibrosis. To our knowledge, this is the first case reported of this unique association.

## 1. Introduction

Dermatomyositis (DM) is an autoimmune mediated inflammatory myopathy characterized by proximal muscle weakness and classic dermatologic findings including violaceous pigmentation (heliotrope sign) [1]. Though primarily idiopathic, DM has been associated with underlying malignancies in a paraneoplastic manner [1–4]. To our knowledge, this is the first case report with DM associated with secondary myelofibrosis following polycythemia vera.

## 2. Case Report

A 69-year-old male with a 15-year history of polycythemia vera (PV), JAK2 V617 positive, presented with a chief complaint of worsening weakness over 4 days. His bilateral upper and lower extremities became spontaneously weak without pain, and he reported difficulty ambulating. He also noted significant dysphagia and mild hoarseness, urinary retention, and constipation.

The patient's PV had previously been managed with hydroxyurea which was discontinued 2 months prior due to pancytopenia. Patient denied any fevers or night sweats; however, he did endorse weight loss and fatigue due to poor oral intake secondary to dysphagia.

On presentation, the patient was vitally stable. Physical exam was significant only for marked proximal, bilateral, upper, and lower extremity weakness. Splenomegaly was absent. Patient was also noted to have a violaceous rash across his forehead.

**Figure 1:**
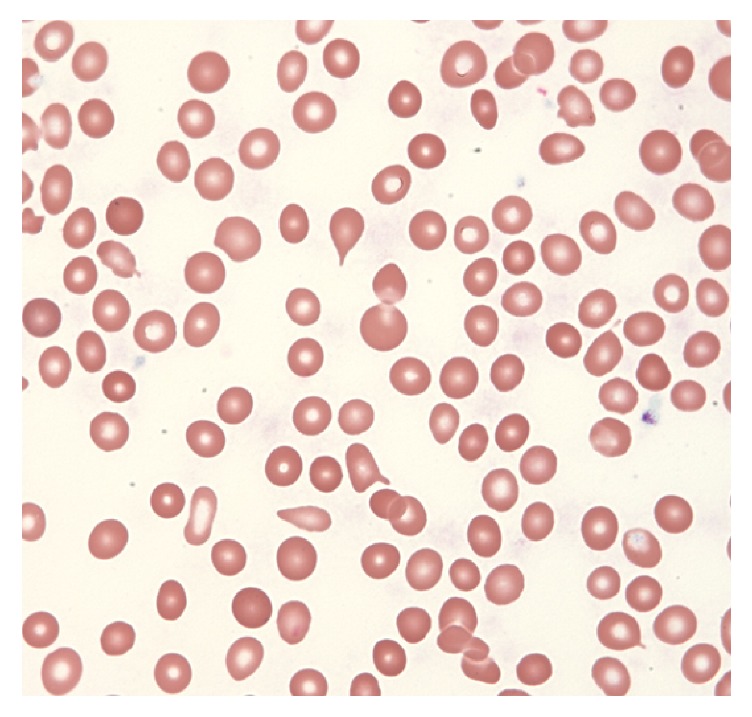
Peripheral blood smear with pancytopenia and teardrop red blood cells (Wright 400x).

Initial complete blood cell count was significant for leukopenia with left shift (WBC 3.1 g/dL), normocytic anemia (Hgb 7.0 g/dL, MCV 89.9 fL), and a normal platelet count. Peripheral blood smear noted teardrop red blood cells and circulating nucleated red cells (Figure 1). ESR was mildly elevated (26) as was CRP (8.5) indicative of an inflammatory state. Labs supportive of myositis included elevated creatinine kinase (6201 U/L), aldolase (42 U/L), LDH (747 U/L), and myoglobin (2843 mcg/L).

**Figure 2:**
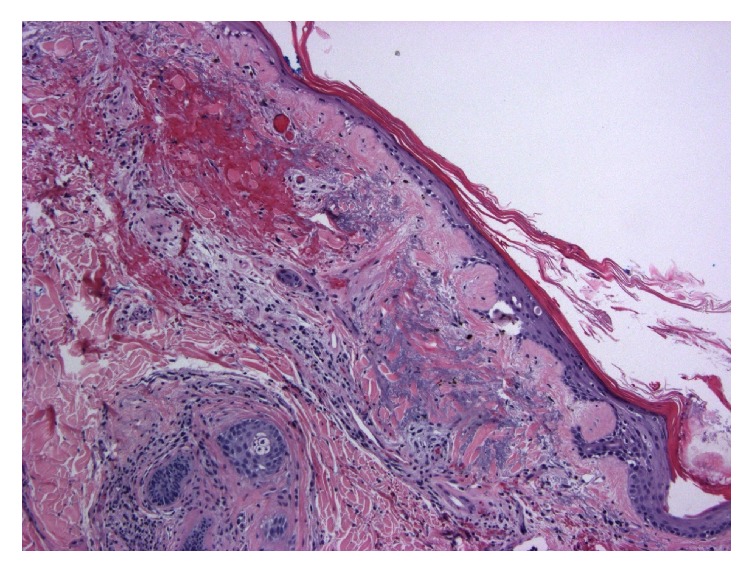
Epidermal atrophy with prominent basement membrane (H&E 100x). Dermal edema with blue mucin and mild perivascular mononuclear infiltrate (mainly lymphocytes).

**Figure 3:**
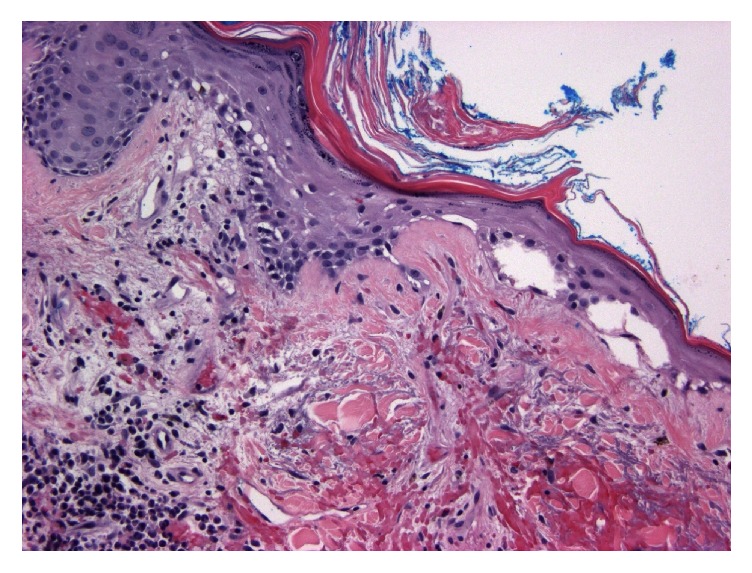
Vacuolar change due to basal cell degeneration (H&E 200x). Civatte bodies at the papillary dermis. Prominent perivascular lymphocytes at left lower corner.

**Figure 4:**
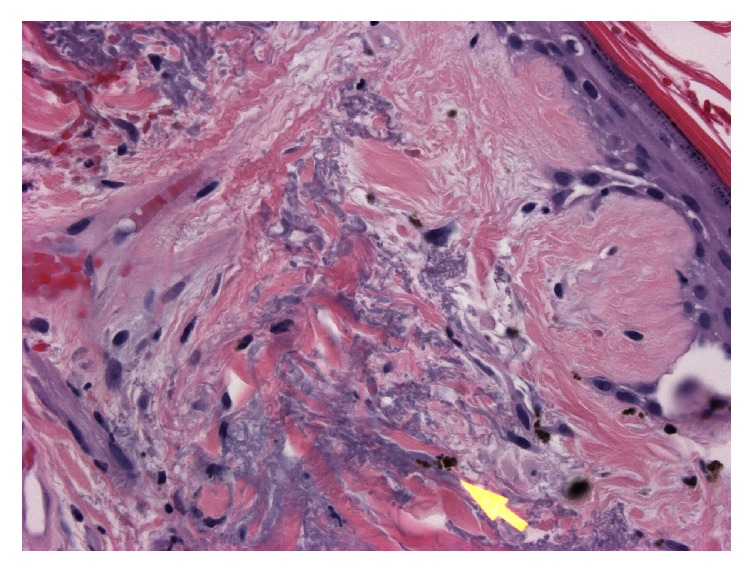
Melanin incontinence into dermis (yellow arrow) (H&E 400x).

MRI with contrast of the spine and brachial plexus were positive for edema of all visualized muscle groups compatible with myositis. Muscle biopsy was attempted but was nondiagnostic and open surgical biopsy was considered. Due to ongoing comorbid conditions, less invasive skin biopsy was performed instead. Pathology at the site of violaceous rash on forehead was consistent with dermatomyositis (Figures 2–4).

Given age and dysphagia at presentation, patient was at high risk for underlying neoplastic process and a malignancy screen was performed. Serum paraneoplastic antibody panel was positive only for striational antibody with titer 1 : 960. CSF paraneoplastic antibody was negative. CT with contrast of the chest, abdomen, and pelvis was negative for splenomegaly and masses.

**Figure 5:**
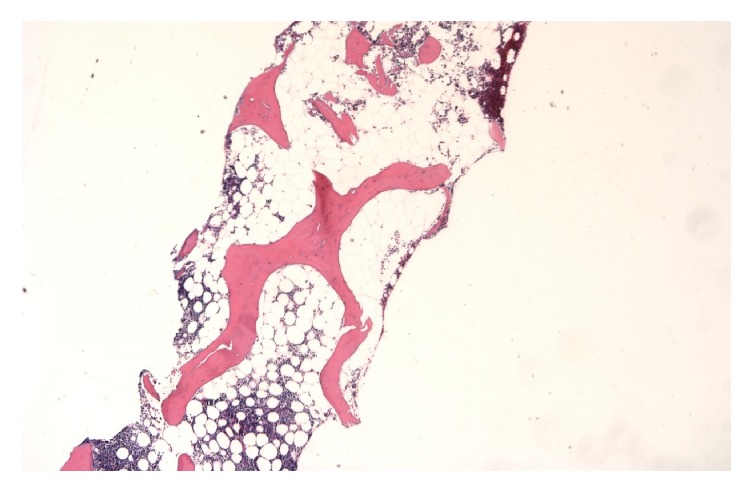
The bone is normocellular with megakaryocytic hyperplasia and moderate fibrosis (bone marrow, left posterior iliac crest, clot section, and trephine core biopsies, 100x).

**Figure 6:**
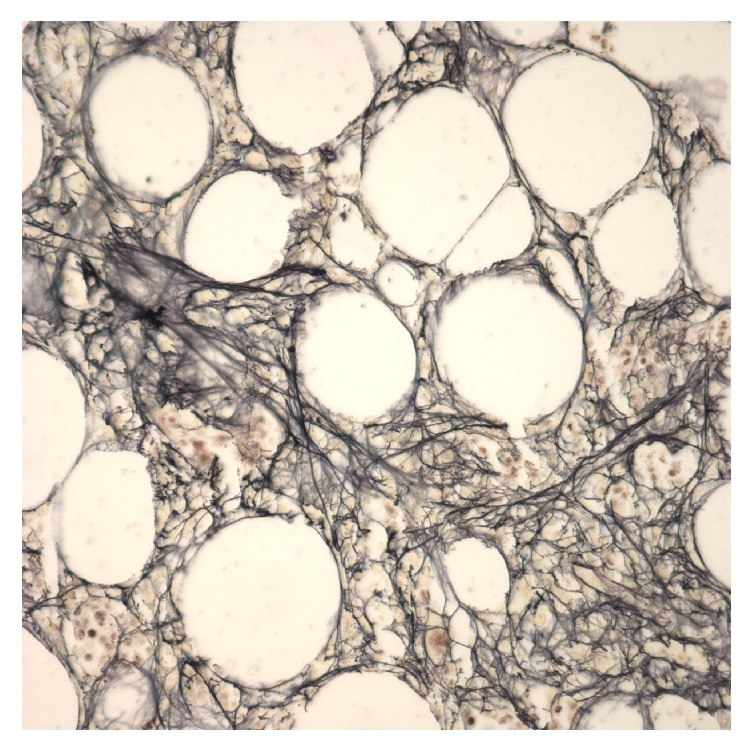
Marrow reticulin fibers moderately increased with storage iron present (bone marrow biopsy, left posterior iliac crest, and reticulin stain, 400x).

Due to history of PV and declining blood counts, a bone marrow biopsy was performed. Immunohistochemical staining with CD34, CD71, Factor VIII, and MPO was performed. Pathologist review reported normocellular marrow with megakaryocyte hyperplasia, mild decrease in erythroid precursors, and moderate fibrosis (Figures 5-6). Results were consistent with postpolycythemic myelofibrosis (MF). Staging yielded IPSS 2 (INT-1) and DIPSS 3 (INT-2), high intermediate to high risk MF. Given the temporal relationship of symptom onset with conversion of PV to MF, the patient was considered to have dermatomyositis associated with secondary MF.

Original treatment included prednisone 40 mg daily and azathioprine 100 mg daily; however, symptoms progressed and pancytopenia worsened over 2 weeks. Azathioprine and prednisone were discontinued, and intravenous immunoglobulin (IVIG) was administered. 1 day after treatment, the patient developed shortness of breath and was found to have a pulmonary embolus with right peroneal deep vein thrombosis. IVIG was subsequently discontinued and the patient was treated with warfarin. Prednisone was restarted at an increase of 60 mg daily and azathioprine was restarted at 100 mg daily. The patient experienced gradual resolution of weakness but required G-tube placement for continued dysphagia.

At a six-month follow-up patient reported resolving weakness with regain of ambulation and swallowing capacity. At this time, prednisone is gradually being tapered. Postpolycythemic MF is currently managed with surveillance as patient is not transfusion dependent and is not a transplant candidate given poor performance status.

## 3. Discussion

Dermatomyositis (DM) is an autoimmune mediated inflammatory myopathy characterized by proximal muscle weakness and classic dermatologic findings including violaceous pigmentation (heliotrope sign). Definitive diagnosis of DM requires a skin or muscle biopsy in the setting of clinical disease [1].

While the majority of DM is idiopathic in etiology, 15–30% of adult onset DM is associated with malignancy [2, 3]. Risk factors of underlying malignancy in the setting of adult onset DM include older age at disease onset, dysphagia, evidence of capillary damage on muscle biopsy, and cutaneous leukocytoclastic vasculitis [5–7]. While ovarian and lung cancers are most frequently identified with DM, hematologic malignancies have also been associated, most commonly B-cell lymphoma, T-cell lymphoma, and myelodysplastic syndrome [8–11]. There have also been individual cases of primary myelofibrosis associated with DM [4, 12, 13]. To our knowledge, this is the first case of secondary myelofibrosis that has been associated with dermatomyositis.

Autoimmunity is a common theme of both DM and MF. DM is a known autoimmune inflammatory myopathy and MF is associated with increased autoantibody production and circulating immune complexes [4, 12]. Additionally, the association between autoimmune disorders and idiopathic MF has been reported in 10–14% of cases [14–17]. This case of postpolycythemic MF presents the possibility of an immune paraneoplastic mechanism associating secondary MF and DM. Regenerating muscle cells in myositis have been observed to express antigens similar to cancer tissue [1, 18]. In the setting of hematologic malignancy, a paraneoplastic immune response may inadvertently target muscle tissue leading to DM.

The differential diagnosis of this case includes hydroxyurea induced DM-like eruption. Prior case reports have shown development of DM while on hydroxyurea and resolution of DM after cessation of the medication [19, 20]. Given that this patient stopped hydroxyurea 2 months prior to the onset of DM, hydroxyurea induced DM is a less likely diagnosis.

Immunosuppression with concurrent treatment of the underlying malignancy is indicated in the setting of paraneoplastic DM. First-line immunosuppressive therapy often includes prednisone. Prior cases of primary myelofibrosis have shown response to azathioprine and prednisone regimens [12]. However, as seen in this case, neoplastic DM may demonstrate poorer response to treatment than DM in the absence of cancer [1]. High dose intravenous steroids have been documented as a potential second-line therapy [21]. IVIG has also been shown to be beneficial in resistant cases of DM [22, 23].

Prior case reports have documented resolution of DM after treatment of underlying neoplasm [24, 25]. Unfortunately, given the poor clinical condition many patients with paraneoplastic DM present with, surgical resection or chemotherapy may not be tolerated.

An alternative therapeutic target may be the Janus Kinase (JAK) 2 receptor with inhibitors such as ruxolitinib or tofacitinib. JAK2 activating mutations including V617F have been found in multiple myeloproliferative neoplasms such as PV, essential thrombocytopenia, and MF [26]. JAK kinase overactivity has also been noted in several autoimmune disorders including rheumatoid arthritis, systemic lupus erythematosus, and other autoantibody-driven diseases [27]. Given its activity in both autoimmune and myeloproliferative processes, JAK2 inhibition would be a reasonable target in paraneoplastic DM. Case reports documenting concurrent resolution of dermatomyositis and post-PV MF after use of ruxolitinib have been previously reported [28].
